# Levels of Selected Matrix Metalloproteinases, Their Inhibitors in Saliva, and Oral Status in Juvenile Idiopathic Arthritis Patients vs. Healthy Controls

**DOI:** 10.1155/2019/7420345

**Published:** 2019-10-28

**Authors:** Agnieszka Kobus, Joanna Bagińska, Joanna Łapińska-Antończuk, Sławomir Ławicki, Anna Kierklo

**Affiliations:** ^1^Department of Dentistry Propaedeutics, Medical University of Bialystok, ul. Szpitalna 30, 15-295 Bialystok, Poland; ^2^Department of Integrated Dentistry, Medical University of Bialystok, Bialystok, Poland; ^3^Department of Population Medicine and Civilization Disease Prevention, Medical University of Bialystok, Bialystok, Poland

## Abstract

**Aims:**

Matrix metalloproteinases (MMPs) are a group of calcium-dependent zinc-containing proteinases acting both physiologically and in pathological conditions. The aim of this study was to evaluate the concentration of MMP-2, MMP-8, and MMP-9 and their inhibitors TIMP-1 and TIMP-2 of unstimulated whole saliva (UWS) in correlation with the oral health in juvenile idiopathic arthritis (JIA) children.

**Methods:**

The study population comprised 34 JIA patients and 34 age- and sex-matched controls (C). They were divided into two groups: with mixed dentition (MD) and with permanent dentition (PD). Dental caries (DMFT/dmft), unstimulated salivary flow rate (SF), and gingival inflammation (Gingival Index (GI) and Papilla Bleeding Index (PBI)) and oral hygiene (Simplified Oral Hygiene Index (OHI-S)) indices were evaluated. Saliva samples were tested with the enzyme-linked immunosorbent assay (ELISA) for MMP-2, MMP-8, MMP-9, TIMP-1, and TIMP-2. Data were statistically analysed with the Mann–Whitney *U* test and Spearman's rank correlation (*p* < 0.05).

**Results:**

There were no differences in dental hygiene or dental and periodontal status between the JIA and C groups. The MMP-9 concentration was higher in the whole JIA group compared with C (*p*=0.005) and JIA MD groups (*p*=0.038). A positive correlation of MMP-2 with the OHI-S index and a negative correlation of MMP-2 with SF were found in JIA. MMP-9 and its tissue inhibitor TIMP-1 had a positive mean correlation with the GI. A high correlation of MMP-8 with the number of decayed teeth (D) in JIA MD patients (*p*=0.037) was revealed. In the JIA-PD patients, there was a positive correlation of MMP-2, -8, and -9 levels with gingival inflammation indices and a negative correlation of MMP-2 and 8 with the SF.

**Conclusions:**

Despite a comparable clinical oral status of affected and unaffected children, in the JIA patients, a statistically significantly increased level of MMP-9 was found. In reference to the periodontal status, the role of MMPs increased in children with permanent dentition, whereas in reference to dental caries, the period of mixed dentition (MD) was critical.

## 1. Introduction

Juvenile idiopathic arthritis (JIA) is an autoimmune inflammatory disease in children under 16 years of age having symptoms persisting for more than six weeks. Juvenile idiopathic arthritis (JIA) usually comprises not one disease but several disorders. Females are much more frequently affected by almost all types of JIA [1]. The etiopathogenesis of JIA is not entirely understood. Among the possible etiologic factors of JIA are bacterial (*Chlamydophila pneumoniae*, *Mycoplasma pneumoniae*, and *Campylobacter jejuni*) and viral (hepatitis B virus and Epstein–Barr virus) infections, mental trauma, genetic factors such as the contribution of HLA tissue compatibility genes and primary deficiencies in components C1–C4 of the complement system, and autoimmune mechanisms disturbing the metabolism and the excretion of products produced during the inflammation process [2]. The pathogenesis of the disease consists in the abnormal functioning of mechanisms controlling the immune system.

The oral manifestation of JIA may present a wide range of symptoms. Joint inflammation destroys the cartilage, influences bone growth, and indicates bone destruction [3–5]. These alterations may lead to impaired mandibular growth. In 25% to 75% of JIA patients, a temporomandibular joint was involved [6]. These features cause limited mouth opening with progressive open bite, retrognathia, microgenia, and bird-like appearance [7, 8]. The restricted mouth opening and alterations in the masticatory function initiate difficulties in patients' oral hygiene and dental treatment [9]. An increase in caries and periodontal disease incidence was reported [3, 10, 11]; however, there are also studies showing similar caries levels in JIA and healthy children [12]. Another aspect influencing the oral health are medications used to control the inflammation in JIA patients. Sugar-based nonsteroidal anti-inflammatory drugs administered to young children increase the risk of dental caries and, when they are sucked or chewed, may cause soft tissue ulceration and dental erosion [13]. Corticosteroids used in JIA treatment may affect intraoral wound healing and increase the risk of infection [9, 13].

Matrix metalloproteinases (MMPs) are a group of calcium-dependent zinc-containing proteinases acting both physiologically and in pathological conditions. At present, 34 types of genetically different but structurally similar groups of metalloproteinases are known. They include among others the subgroups of collagenases, i.e., MMP-1, -8, -13, causing decomposition of collagen type 1 and gelatinases decomposing degraded collagen, e.g., MMP-9 and MMP-2. Metalloproteinases may degrade almost all components of the intercellular substance and basement membrane. They also control cellular and inflammatory processes by a limited proteolysis of bioactive substances (enzymes, chemokines, cytokines, growth factors, components of the complement system, receptors, etc.) [14]. MMPs are produced by the majority of normal cells, among others, fibroblasts, mast cells, osteoblasts, odontoblasts, dendritic cells, microglia cells, smooth muscle myocytes, keratinocytes, and endothelial cells. These enzymes are also secreted by inflammatory cells, macrophages, T-lymphocytes, monocytes, neutrophils, and eosinophils [7, 9]. The secretion and the activity of metalloproteinases under physiological conditions is controlled by endogenous activators and inhibitors—tissue inhibitors (TIMPs) and serine protease inhibitors [13]. In tissues, the MMP activity is controlled by tissue inhibitors of metalloproteinases (TIMPs 1–4). The mechanism of action of TIMP is based on the inhibition of proenzyme activation or the inactivation of an active enzyme by forming the TIMP-MMP complex [14, 15].

It was shown in numerous research studies that the MMPs level in blood, serum, and oral fluids may be increased due to various diseases or systemic conditions, i.e., hypertension, heart diseases, diabetes, rheumatic disease, and pregnancy [16–20]. It was proven that the concentrations of MMPs, in particular MMP-8 and MMP-9, in sick persons were higher in oral fluids (saliva and gingival pocket fluid) than in serum, blood, or articular fluid [20]. In patients with high blood pressure, an increased level of MMP-8 and lysozyme in saliva and an increase in the MMP-8/TIMP-1 ratio were observed. Diabetic patients had a twice higher MMP-8 level and a three times higher MMP-9/TIMP-1 ratio. Muscular and articular diseases were associated with an increase in IL-1beta and MMP-8 concentrations and MMP-8/TIMP-1 ratio [16].

The role of some salivary MMPs in the degradation of marginal periodontium was confirmed and was found to be highly probable in the degradation of dental hard tissues. Increased MMP-8 and MMP-9 levels were observed in advanced periodontal disease compared with healthy subjects; with the discovery of red complex periodontal pathogens (e.g., *Porphyromonas gingivalis* and *Treponema denticola*), these biomarkers are used for a more detailed classification of the periodontal disease [14, 21, 22]. Tissue metalloproteinase inhibitors (TIMPs) -1, -2, -3, and -4 are the main physiological inhibitors of MMPs and influence their activity also in periodontal diseases. MMP-8 is the strongest biomarker of alveolar bone destruction correlating with clinical and radiological symptoms such as depth of pockets, loss of attachment, and bleeding in probing [21, 22]. By testing the MMP-8 and TIMP-1 levels and the MMP-8/TIMP-1 ratio, individuals with periodontal disease may be differentiated from subject in the control group. A considerable increase in the MMP-8 level and the MMP-8/TIMP-1 ratio was more frequently observed in the course of generalised aggressive periodontal bone loss than in the local form [14]. The MMP-8 level in saliva changes in response to applied treatment. A considerable reduction of the MMP-8 content was observed after removing dental deposits and smoothing the root surface, which correlated with clinical parameters. The activity of MMP-8 is controlled by the expression of its genes, the conversion of zymogen to an active form, and the specific inhibitors.

Shimada et al. investigated the distribution of MMPs in physiological and carious dentine and found a high MMP-8 level in the outer layer of caries (dentine infected by bacteria), with the simultaneous lower content in the inner layer (demineralised, but not yet subject to degradation) [23]. They associated this fact with the inflow of salivary MMPs into the cavity. Metalloproteinases are also produced by odontoblasts and participate in formation, physiology, and pathology of dentinal lesions. Their inactive forms are closed within the dentine after its mineralisation. Under the influence of dentine demineralisation in the acid environment, they are activated and may participate in the carious process, particularly important is MMP-8 which has an affinity to the collagen type 1 (main component of the extracellular substance of dentine) [24]. Also Nascimento et al. noticed an increase in the activity of salivary MMPs in active caries patients compared with chronic caries patients. They also described a gradual increase in the MMP activity with increasing cavity depth [25].

The contribution of MMPs to the etiopathogenesis of rheumatoid arthritis (RA) was widely discussed in the literature. An increased level of inflammatory mediators in saliva and in pocket fluid in RA patients examined by Arvikar et al. [20] was independent of the occurrence of clinical lesions in the gingiva and in the periodontium. These authors also observed that MMP-8 and MMP-9 concentrations in the pocket fluid were four times higher than those in the serum [20]. So it is possible that oral tissues may be a site of extra-articular inflammation in inflammatory arthritis patients [20]. In the literature, there are relatively few reports on the influence of MMPs and their tissue inhibitors on the oral status of JIA patients. Therefore, the aim of the present study was to evaluate the concentration of MMP-2, MMP-8, and MMP-9 and their inhibitors TIMP-1 and TIMP-2 of unstimulated whole saliva (UWS) in correlation with oral health in JIA children including mixed and permanent dentition.

## 2. Materials and Methods

This study was approved by the Bioethical Committee of the Medical University of Bialystok, Poland (No. R-I-002/53/2008 and No. R-I-002/494/2015). An informed written consent was obtained from the parent(s) or guardian(s) and a verbal consent was given by the patients after explanation of the nature, purpose, and potential risks of the study.

### 2.1. Subjects

The subjects participating in this study were patients of the Outpatient Clinic at the Department of Paediatrics and Developmental Disorders, Medical University of Bialystok, Poland. This is the only paediatric rheumatology clinic in northeastern Poland. The minimum sample size was assessed to be 24 children based on the following assumptions: the number of children aged 6 to 18 within the range of activity of the Department of Paediatrics and Developmental Disorders, 900,000; the prevalence of JIA 0.00065, the 95% confidence level; and the 1% measuring error. Diagnosed JIA was the criterion for inclusion in the study. The exclusion criteria comprised the presence of another chronic disease, therapy using medication interfering with the salivary secretion within the last year, and the onset of menstruation for females. All patients were examined by the same physician according to the ILAR classification [26]. The subjects were recruited successively at routine follow-up visits. The total number of patients invited to the study was 61; however, 7 parents refused to give consent to the participation of their children and 20 patients were excluded from the study. The subjects were divided into two subgroups: with mixed dentition (MD) and with permanent dentition (PD).

### 2.2. Controls

Children not affected by JIA (C), with year of birth, gender, and ethnicity matching with the subjects, were recruited from a local dental practice. For each subject, at least two potential controls were identified and one of them was randomly selected. In the case of refusal or failure to meet the inclusion criteria, the next child from the list was invited to participate in the study. The subjects met all of the following eligibility criteria: good health and no systemic illness or hospitalization within the last two years, no known history of chronic disease and no medication or hormones interfering with salivary secretion within the last year, and for females, the onset of menstruation. Finally, one matching control for each subject was found.

### 2.3. Oral Examination/Clinical Assessment

All patients were asked questions about the subjective oral dryness and angulitis, the frequency of consumption of sweets, and the history of occlusal abnormalities in the family. All clinical examinations were performed by the same qualified dental surgeon (AK) under standardized conditions, in a dental chair, with the use of portable equipment provided with artificial light, suction device, and compressed air. All examinations were conducted by means of diagnostic dental tools (plane mirror, clinical probe, and periodontal probe). In accordance with the World Health Organization criteria, the level of dental caries was determined using the DMFT index (decayed, missing, or filled teeth in the permanent dentition) based on the clinical examination without a dental X-ray in children with permanent and mixed teeth and using the dmft index (decayed, missing, or filled teeth in the primary dentition) in children with mixed dentition [27]. White spot lesions were excluded. The gingival status was assessed using the Gingival Index (GI) [28] and Papilla Bleeding Index (PBI) [29]. GI was coded as follows: 0, no gingivitis; above 0 to 1, mild gingivitis; above 1 to 2, moderate gingivitis; and above 2 up to 3, severe gingivitis. PBI was calculated by dividing total bleeding in probing interdental papilla by the number of examined interdental papilla. The Simplified Oral Hygiene Index (OHI-S) was used to determine the level of oral hygiene [30]. It was assumed that the OHI-S index fluctuated between 0 and 6 where 0–2 meant good oral hygiene, 2–4 satisfactory oral hygiene, and 4–6 bad oral hygiene.

All of the examined patients were offered dental treatment, but only a small percentage of them agreed. A routine hygienic procedure as well as the caries and preventive treatment was performed. Antibiotics were used only in the group of patients with a high risk of bacteraemia.

Before the commencement of the study, a calibration for caries by double examination of 10 children aged between 6 and 15 at the interval of one week was performed. The intra-examiner agreement (unweighted Cohen's kappa coefficient) was 0.89 for primary dentition and 0.92 for permanent dentition.

### 2.4. Saliva Collection

The subjects were instructed to refrain from consuming food and beverages, except water, for two hours before saliva collection. For saliva collection, each participant was seated in a chair in a well-ventilated room and protected from gustatory and other stimulations. Resting whole saliva samples were collected in plastic tubes and placed on ice for 15 min, under the control of one dentist (AK), by the passive spitting method, between 8:00 and 10:00 AM to minimize the circadian rhythm effects [31, 32]. The volume of each sample was measured with a pipette calibrated in 0.1 ml units. The salivary flow rate (SF) was determined from the obtained volume divided by the time needed for sample collection.

### 2.5. Biochemical Analyses

Saliva samples were collected from each patient, centrifuged with 100 rpm for 15 min, and stored at −85°C until assayed. The tested parameters were measured with enzyme-linked immunosorbent assay (ELISA) (MMP-2, MMP-8, MMP-9, TIMP-1, and TIMP-2—Quantikine Human Immunoassay, R&D Systems) according to the manufacturer's protocols. Duplicate samples were assessed for each patient in ELISA.

The intra-assay coefficient of variation (CV%) of MMP-2 was found to be 3.8% at a mean concentration of 11.20 pg/mL, SD = 0.42, and TIMP-2 was found to be 6.0% at a mean concentration of 2.90 pg/mL, SD = 0.173. MMP-9 was found to be 1.9% at a mean concentration of 2.04 ng/mL, SD = 0.039, and TIMP-1 was found to be 3.9% at a mean concentration of 1.27 ng/mL, SD = 0.05. MMP-8 was found to be 5.0% at a mean concentration of 3.61 ng/mL, SD = 0.182.

The inter-assay coefficient of variation (CV%) of MMP-2 was found to be 6.6% at a mean concentration of 11.1 pg/mL, SD = 0.738, and TIMP-2 was found to be 6.7% at a mean concentration of 2.79 pg/mL, SD = 0.188. MMP-9 was found to be 7.8% at a mean concentration of 2.35 ng/mL, SD = 0.184, and TIMP-1 was found to be 3.9% at a mean concentration of 1.28 ng/mL, SD = 0.05. MMP-8 was found to be 4.2% at a mean concentration of 3.53 ng/mL, SD = 0.147.

### 2.6. Statistical Analysis

The statistical analysis was conducted using the STATISTICA 10.0 PL program. A preliminary statistical analysis (chi-square test) revealed that the distribution of tested parameter levels failed to follow normal distribution. Consequently, the Mann–Whitney *U* test was used for a statistical analysis of differences between JIA patients and control groups. The data were presented as a median and a range. Spearman's rank correlation was used for the purpose of analysis of correlations. Statistically significant differences were defined as comparisons resulting in *p* < 0.05.

## 3. Results

The final study population comprised thirty-four subjects diagnosed with JIA (aged 6 to 18 years, 64.7% of females) and a corresponding number of controls. The mixed dentition (MD) subgroup comprised 15 patients (aged 6 to 10 years) and the permanent dentition subgroup (PD) 19 patients (aged 11 to 18 years). In Table 1, age and sex of the participants and the disease duration are presented in detail, including the type of dentition. The mean disease duration was 4.62 years. A detailed dental oral status of JIA children, including mixed and permanent dentition, is shown in Table 2. We did not find any differences in dental hygiene or dental and periodontal status between the JIA children and the control group. SF was significantly lower in the JIA group as compared with the C (*p*=0.027). The classification of JIA children according to the type of dentition revealed a significantly lower SF in the PD JIA group compared with the controls (*p*=0.019).

Table 3 shows mean concentrations of MMP-2, -8, and -9 and their inhibitors TIMP-1 and TIMP3-2 in unstimulated saliva in JIA children and controls (C), including mixed and permanent dentition. The MMP-9 concentration was more than twice higher in JIA children compared with the control group (*p*=0.005). This pattern was also found in JIA children with MD (*p*=0.038). There were no significant differences in MMP-2, MMP-8, TIMP-1, and TIMP-2 concentrations in UWS between the JIA and C groups either in the whole sample group or after the division of the children according to the type of dentition.

In the course of JIA, a positive mean correlation of MMP-2 with the OHI-S index and a negative mean correlation of MMP-2 with SF were found. MMP-9 and its inhibitor TIMP-1 had a positive mean correlation with the GI, Table 4. In the control group, no significant correlations with clinical oral parameters were found. The division of patients according to the type of dentition showed a high correlation of MMP-8 with the number of decayed teeth (D) in JIA MD patients (*p*=0.037). In this population (JIA MD), a high correlation of the TIMP-1 inhibitor with the PBI and a high negative correlation of the TIMP-1 inhibitor with the salivary secretion rate SF were also found, Table 5. In addition, in JIA MD patients in whom the hyposalivation was found, the TIMP-1 concentration was significantly higher compared with JIA MD patients with normal salivary secretion (*p*=0.008). In JIA-PD patients, a mean correlation of MMP-2 with the GI and a high correlation of MMP-2 with the PBI were observed. In this children group, a mean correlation of MMP-8 with the GI and a negative mean correlation of MMP-8 with SF were also found. MMP-9 also had a mean correlation with the GI in the permanent dentition patients. In the control group, only a negative mean correlation of TIMP-2 with SF in the permanent dentition (PD) population was noted, Table 5.

## 4. Discussion

The concentration of metalloproteinases in blood, serum, and articular fluid in JIA patients and their diagnostic potential were widely discussed in the literature [33–35]. However, only a few papers considered the MMP concentration in the saliva of JIA patients [36]. The saliva is an easily accessible, noninvasively collected diagnostic material, which is particularly important in small patients [37]. Therefore, we think that the research we conducted is valuable and has contributed substantial knowledge of the role of MMPs in the development of oral lesions in autoimmune disease patients. In the sample population, there was no difference in oral status (caries level, oral hygiene, and periodontal condition) between the study and control groups, which may be explained by a high level of dental caries and the negligence in dental hygiene in Polish children and adolescents [9]. The control group presented an average level of dental caries for the Polish population. It could be expected that in the population with a low caries prevalence and experience, the differences between the study and controls were present. Also, Miranda et al., when analysing the influence of rheumatic disease and its treatment on the periodontal status, did not find any significant differences in oral clinical status in the course of JIA as compared with the control group [4].

A biochemical examination of saliva showed a higher mean level of MMP-2, MMP-8, and MMP-9 and TIMP-1 inhibitor in the saliva of JIA patients compared with the C group; however, a statistically significant difference was demonstrated only in the case of MMP-9 in all patients as well as in the group of children with mixed dentition (JIA MD). In the permanent dentition group, there were no statistically significant differences. The reports from the literature concerning the level of MMPs in JIA are ambiguous. In the research on the level of antioxidants and MMPs in the saliva of JIA patients, Brik et al. found a lower MMP-9, MMP-2, and MMP-3 level compared with healthy individuals [36]. It concerned patients treated and not treated with anti-TNF medications as well as patients with active and inactive disease. The authors associated this fact with a reduction of calcium contained in the saliva of JIA patients and an increase in its antioxidant activity. On the contrary, Miranda et al. [4] showed a similar MMP-8 level in the gingival pocket fluid in the course of JIA compared with the control group; however, a difference in the material collection methodology (gingival pocket vs. unstimulated oral saliva) makes it impossible to directly compare their results with our findings.

In the present study, a positive mean correlation of MMP-2 with the oral hygiene status (OHI-S) and a positive mean correlation of MMP-9 and its inhibitor TIMP-1 with the gingivitis (GI) in the entire examined group were obtained, whereas in the control group, the oral hygiene level and the presence of gingival inflammation did not influence the MMP level. MMP-2 and MMP-9 are gelatinases which are active primarily in relation to degraded collagen. A transition of proenzymes into an active form requires a low pH which may be generated by bacteria present in the oral cavity in the course of the conversion of sugars supplied in the food. The buffer capacity of saliva responsible for maintaining an optimal pH in the oral cavity depends on the salivary secretion rate, and JIA patients had a clearly lower unstimulated SF compared with the C group. A high concentration of MMPs and their inhibitors in saliva was probably not caused by an increased release of analysed proteins but only by a reduced saliva production by salivary glands affected in the course of JIA. The damage of salivary glands in the course of JIA was also suggested by Brik et al. [38].

It is noteworthy that, in this study, MMP-2, MMP-8 and -9 significantly correlated with the gingival inflammation indices in JIA-PD patients. Moreover, a high correlation of the TIMP-1 inhibitor with the bleeding index (PBI) in mixed dentition patients was found. It is believed that the MMP-8 level and the MMP-8/TIMP-1 ratio strongly correlate with clinical parameters in the periodontal disease such as the depth of gingival pockets or the bleeding in probing. A reduction in the MMP-8 level to a physiological level contributes to the subsidence of inflammation by the conversion of anti-inflammatory chemokines and cytokines, which suggest also a defensive role of MMP-8 in the periodontal inflammation [21, 22]. The correlation of MMPs with the gingival inflammation shown by us may prognosticate pathological lesions within the oral cavity increasing with the age and progression of JIA.

With reference to dental caries, a high positive correlation of MMP-8 with the number of teeth with active caries (D) in the mixed dentition group (JIA MD), so in the period of eruption of permanent teeth, was found. In the JIA-PD group, also a positive correlation with D (without statistical significance) was shown. There were no such relationships in the control group despite the fact that the children of the C-PD group had more teeth with active caries than the JIA children with permanent dentition (JIA-PD). MMP-8 is the primary collagenase detected in the gingiva and in the oral fluids. Its main source is polymorphonuclear neutrophils, but it may be also produced by odontoblasts in the course of caries [39]. The demonstrated correlation of MMP-8 with the number of teeth with active caries may be both a cause and an effect of an acute course of caries resulting in a faster destruction of hard tissues in JIA children. As in this study, only the presence of carious lesions without the assessment of their progression was noted, it is not possible to determine whether such influence occurred. However, a high positive correlation of MMP-8 with the number of teeth with active caries in mixed dentition proves the need of enhanced caries prevention in JIA patients from the onset of the disease.

### 4.1. Limitations

The present study was conducted on a small group, which is to be regarded as a limitation. The numerical strength of the sample group resulted from a rare occurrence of JIA. Another limitation was that we assessed only the saliva without the serum and the gingival cervical fluid. Noteworthy is that the saliva offers distinctive advantages, namely, it may be noninvasively collected, which is very important in children, it does not require special equipment for collection and storage, and it does not clot. Therefore, analytes such as MMPs related to autoimmune diseases and oral diseases could be easily implemented in diagnostic applications.

## 5. Conclusions

The obtained results indicate that—with reference to the periodontal status—the role of MMPs increases in adolescents (JIA-PD) compared with younger children (JIA MD), whereas in reference to dental caries, the period of eruption of mixed dentition is critical. JIA patients should strictly adhere to the removal of dental plaque as the main cause of gingival inflammations and dental caries. It is very important in view of the fact that they have impaired mechanisms of natural oral cavity cleaning due to salivary secretion disorders. Due to a small sample size, further insightful observations of the MMPs level in saliva and of their influence on clinical oral status in the course of JIA in a greater sample are needed.

## Figures and Tables

**Table 1:** Disease duration and demographic characteristics in JIA children and controls (C) including the type of dentition.

Demographic characteristic			Mixed dentition		Permanent dentition		Total	
			JIA *N* = 15	C *N* = 15	JIA *N* = 19	C *N* = 19	JIA *N* = 34	C *N* = 34
Disease duration (years)		Mean (SD)	3.15 (2.61)		5.71 (3.79)		4.62 (3.53)
		Min-Max	0.25–8		0.17–13		0.17–13
Age (years)		Mean (SD)	7.47 (1.46)	8.53 (2.35)	15.95 (2.17)	15.89 (2.28)	12.29 (4.57)	12.64 (4.35)
		Min-max	6–10	6–13	11–18	11–18	6–18	6–18
		*p* value	0.384		1.0		0.74	
Sex	Female	Mean (%)	10 (66.67%)	10 (66.67%)	11 (57.90%)	11 (57.90%)	21 (61.76)	21 (61.76)
	Male	Mean (%)	5 (33.33)	5 (33.33)	8 (42.10)	8 (42.10)	13 (38.24)	13 (38.24)

*Note.* Mann–Whitney test.

**Table 2:** Oral parameters in JIA children and controls (C) including the type of dentition.

Variables		Total		Mixed dentition		Permanent dentition	
		JIA *N* = 34	C *N* = 34	JIA *N* = 15	C *N* = 15	JIA *N* = 19	C *N* = 19
Decayed	Mean (SD)	1.94 (2.37)	2.47 (3.59)	0.53 (1.06)	0.2 (0.56)	3.05 (2.55)	4.26 (3.96)
	Min-Max	0–10	0–11	0–4	0–2	0–10	0–11
	*p* value	0.69		0.23		0.55	

Missing	Mean (SD)	0.23 (0.92)	0.06 (0.24)	0	0	0.42 (1.22)	0.1 (0.31)
	Min-Max	0–5	0–1	0	0	0–5	0–1
	*p* value	0.61			1.0	0.57	

Filled	Mean (SD)	4.03 (4.63)	3.18 (3.86)	1.47 (2.23)	1.07 (1.71)	6.04 (5.06)	4.84 (4.13)
	Min-max	0–17	0–14	0–7	0–5	0–17	0–14
	*p* value	0.62		0.66		0.62	

Number of caries free individuals		6	9	5	8	1	1

DMFT	Mean (SD)	6.21 (5.49)	5.71 (5.33)	2 (2.36)	1.27 (1.67)	9.53 (4.96)	9.21 (4.54)
	Min-max	0–19	0–17	0–7	0–5	0–19	0–17
	*p* value	0.66		0.66		0.66	

OHI-S	Mean (SD)	0.95 (0.55)	0.85 (0.55)	1.07 (0.42)	0.90 (0.62)	0.85 (0.63)	0.81 (0.50)
	Min-Max	0–2.17	0–2.17	0–1.83	0–2.17	0–2.17	0–1.50
	*p* value	0.45		0.24		0.89	

PBI	Mean (SD)	0.2 (0.31)	0.25 (0.27)	0.09 (0.19)	0.22 (0.32)	0.29 (0.37)	0.27 (0.22)
	Min-max	0–1.17	0–1	0–0.67	0–1	0–1.17	0–0.67
	*p* value	0.23		0.2		0.7	

GI	Mean (SD)	0.25 (0.34)	0.24 (0.27)	0.21 (0.34)	0.19 (0.29)	0.29 (0.34)	0.28 (0.25)
	Min-max	0–1	0–1	0–1	0–1	0–1	0–0.83
	*p* value	0.75		0.92		0.75	

SF (ml/min)	Mean (SD)	0.41 (0.28)	0.51 (0.25)	0.37 (0.30)	0.38 (0.14)	0.43 (0, 27)	0.61 (0.27)
	Min-max	0.04–1.33	0.19–1.17	0.04–1	0.19–0.63	0.19–1.33	0.29–1.17
	*p* value	0.027^*∗*^		0.33		0.019^*∗*^	

*Note.* Mann–Whitney test; ^*∗*^statistically significant.

**Table 3:** Concentrations of metalloproteinases MMP-2, MMP-8, and MMP-9 and their inhibitors TIMP-1 and TIMP-2 in UWS of JIA children and controls (C) including mixed and permanent dentition.

Salivary parameters		Total			Mixed dentition			Permanent dentition		
		JIA *N* = 34	C *N* = 34	*p* value	JIA *N* = 15	C *N* = 15	*p* value	JIA *N* = 19	C *N* = 19	*p* value
MMP-2	Mean (SD)	1.18 (1.82)	0.81 (1.06)	0.54	1.5 (2.23)	0.95 (1.32)	0.41	0.93 (1.43)	0.71 (0.82)	0.83
	Min-max	0–8.62	0–4.78		0–8.62	0–4.77		0–5.54	0–3.68	

MMP-8	Mean (SD)	237.95 (224.17)	198.31 (160.53)	0.73	210.07 (164.68)	172.68 (164.04)	0.21	259.97 (264.34)	218.55 (159.16)	0.76
	Min-max	40.8–908	49.6–752		48.32–590	49.6–576		40.8–908	57.92–752	

MMP-9	Mean (SD)	180.63 (200.69)	85.3 (143.3)	0.005^*∗*^	151.85 (147.42)	64.78 (84.77)	0.038^*∗*^	203.34 (236.01)	101.5 (177.28)	0.07
	Min-max	0–960	0–628.6		0–603.8	0–253.48		0–960	0–628.6	

TIMP-1	Mean (SD)	266.1 (185.17)	211.09 (169.81)	0.227	256.98 (194.36)	220.52 (184.49)	0.68	273.31 (182.64)	203.65 (162.04)	0.3
	Min-max	0–667	0–737		38.5–667	15.5–737		0–609.1	0–527.2	

TIMP-2	Mean (SD)	21.08 (12.1)	23.07 (15.61)	0.77	20.71 (10.95)	26.2 (20.89)	0.74	21.38 (13.23)	20.6 (9.63)	0.87
	Min-max	3.87–50.58	8.41–76.12		7.5–47.3	8.41–76.12		3.87–50.58	10.55–48.76	

*Note.* Mann–Whitney test; ^*∗*^statistically significant.

**Table 4:** Correlations of MMPs and their inhibitors with clinical oral parameters in the course of JIA in the sample population.

Salivary parameters		JIA						C					
		DMFT	D	OHI-S	GI	PBI	SF	DMFT	D	OHI-S	GI	PBI	SF
MMP-2	*r*	0.11	0.005	0.36	0.28	0.26	−0.37	0.08	0.2	−0.12	0.14	0.12	−0.10
	*p*	0.538	0.97	0.036^*∗*^	0.109	0.139	0.033^*∗*^	0.641	0.26	0.485	0.440	0.488	0.576

MMP-8	*r*	0.16	0.14	0.21	0.26	0.26	−0.22	0.11	0.19	−0.04	0.19	0.19	0.18
	*p*	0.353	0.416	0.242	0.134	0.143	0.209	0.522	0.29	0.826	0.275	0.284	0.318

MMP-9	*r*	0.26	0.14	0.13	0.39	0.20	−0.13	−0.15	0.05	−0.24	−0.15	−0.13	−0.22
	*p*	0.133	0.421	0.48	0.021^*∗*^	0.257	0.461	0.404	0.78	0.177	0.398	0.461	0.206

TIMP-1	*r*	0.15	−0.02	−0.01	0.38	0.30	−0.30	0.08	0.24	−0.17	−0.01	−0.05	−0.26
	*p*	0.405	0.9	0.94	0.027^*∗*^	0.080	0.088	0.669	0.17	0.334	0.940	0.759	0.136

TIMP2	*r*	0.15	−0.04	0.09	0.29	0.20	−0.31	−0.15	0.12	−0.31	−0.16	−0.04	−0.25
	*p*	0.402	0.8	0.6	0.092	0.248	0.076	0.406	0.5	0.079	0.375	0.823	0.155

*Note.* Spearman's rank correlation; ^*∗*^statistically significant.

**Table 5:** Correlations of MMPs and their inhibitors with clinical oral parameters in the course of JIA and in the C group in mixed dentition (MD) and permanent (PD) dentition children.

Dentition type		Salivary parameters									
		MMP-2		MMP-8		MMP-9		TIMP-1		TIMP-2	
		*r*	*p*	*r*	*p*	*r*	*p*	*r*	*p*	*r*	*p*
JIA-MD	DMFT	0.49	0.064	0.47	0.075	0.43	0.113	−0.08	0.782	0.25	0.363
	D	0.2	0.48	0.54	0.037^*∗*^	0.42	0.12	0.19	0.49	0.41	0.13
	OHI-S	0.12	0.677	0.03	0.923	0.01	0.975	0.09	0.757	−0.13	0.644
	GI	0.08	0.789	−0.04	0.874	0.15	0.596	0.41	0.130	0.11	0.700
	PBI	0.08	0.770	0.23	0.415	0.43	0.112	0.75	0.001^*∗*^	0.42	0.121
	SF	−0.18	0.532	0.04	0.894	−0.22	0.431	−0.63	0.012^*∗*^	−0.29	0.286

C-MD	DMFT	0.15	0.593	−0.48	0.071	−0.45	0.094	0.21	0.461	−0.26	0.344
	D	0.5	0.06	0.02	0.932	−0.07	0.81	0.21	0.45	0.51	0.054
	OHI-S	−0.06	0.824	−0.03	0.929	−0.11	0.706	−0.06	0.819	−0.12	0.678
	GI	−0.05	0.860	0.07	0.797	−0.02	0.930	0.21	0.452	−0.16	0.570
	PBI	0.13	0.641	0.41	0.134	0.07	0.806	−0.16	0.578	0.10	0.731
	SF	0.15	0.593	0.06	0.829	−0.07	0.801	−0.24	0.388	−0.05	0.854

JIA-PD	DMFT	0.23	0.351	0.17	0.494	0.31	0.193	0.28	0.248	0.22	0.371
	D	0.24	0.31	0.25	0.3	0.04	0.88	−0.24	0.33	−0.11	0.65
	OHI-S	0.39	0.095	0.27	0.266	0.13	0.583	−0.06	0.821	0.11	0.655
	GI	0.59	0.008^*∗*^	0.48	0.036^*∗*^	0.50	0.027^*∗*^	0.38	0.113	0.40	0.087
	PBI	0.60	0.006^*∗*^	0.35	0.138	0.08	0.757	0.06	0.809	0.12	0.620
	SF	−0.44	0.063^*∗*^	−0.46	0.049^*∗*^	−0.02	0.946	−0.03	0.900	−0.35	0.143

C-PD	DMFT	0.08	0.751	0.03	0.914	−0.04	0.866	0.18	0.463	0.01	0.977
	D	0.09	0.71	−0.01	0.98	0.08	0.75	0.36	0.135	0.05	0.83
	OHI-S	−0.31	0.200	−0.06	0.795	−0.35	0.137	−0.18	0.452	−0.45	0.055
	GI	0.29	0.235	0.19	0.425	−0.22	0.357	−0.12	0.626	−0.07	0.767
	PBI	0.11	0.650	−0.09	0.701	−0.24	0.318	0.004	0.985	−0.10	0.676
	SF	−0.39	0.103	0.08	0.758	−0.37	0.118	−0.44	0.061	−0.47	0.04^*∗*^

*Note.* Spearman's rank correlation; ^*∗*^statistically significant.

## Data Availability

The clinical and biochemical data used to support the findings of this study are available from the corresponding author upon request.

## Abbreviations

JIA: Juvenile idiopathic arthritis
MMPs: Matrix metalloproteinases
TIMPs: Tissue metalloproteinase inhibitors
RA: Rheumatoid arthritis
MD: Mixed dentition
PD: Permanent dentition
C: Controls
DMFT index: Decayed, missing, or filled teeth index in the permanent dentition
dmft index: Decayed, missing, or filled teeth index in the primary dentition
GI: Gingival Index
OHI-S: Simplified Oral Hygiene Index
PBI: Papilla Bleeding Index
SF: Salivary flow rate.
